# Comparison of cost effectiveness between video-assisted thoracoscopic surgery (vats) and open lobectomy: a retrospective study

**DOI:** 10.1186/s12962-021-00307-2

**Published:** 2021-08-28

**Authors:** Wei Chen, Zhanwu Yu, Yichen Zhang, Hongxu Liu

**Affiliations:** 1 Department of Thoracic Surgery, Cancer Hospital of China Medical University, Liaoning Cancer Hospital & Institute, No. 44 Xiaoheyan Road, Shenyang, 110042 Liaoning P.R. China; 2 Department of Health Policy and Management, Tulane University, School of Public Health and Tropical Medicine, New Orleans, LA 70112 USA

**Keywords:** Cost effectiveness, Video-Assisted Thoracoscopic Surgery (VATS), Open lobectomy, Lung cancer

## Abstract

**Background:**

Lung cancer is highly prevalent in Chinese population. The association of operative approach with economic burden in these patients remains unknown.

**Objectives:**

This institution-level cohort study aimed to compare the cost-related clinical outcomes and health care costs among patients undergoing video-assisted thoracoscopic surgery (VATS) and open lobectomy, and to investigate the factors associated with the costs.

**Methods:**

This retrospective cohort study included patients who underwent VATS or open lobectomy in a provincial referral cancer center in China in 2018. Propensity score matching (PSM) method was applied to balance the baseline characteristics in VATS lobectomy and open lobectomy group. Clinical effectiveness measures included post-operative blood transfusion, lung infection, and length of stay (LOS). Hospitalization costs were extracted from hospital information system to assess economic burden. Multivariable generalized linear model (GLM) with gamma probability distribution and log-link was used to analyze the factors associated with total costs.

**Results:**

After PSM, 376 patients were selected in the analytic sample. Compared to open lobectomy group, the VATS lobectomy group had a lower blood transfusion rate (2.13% vs. 3.19%, P = 0.75), lower lung infection rate (21.28% vs. 39.89%, P < 0.001) and shorter post-operative LOS (9.4 ± 3.22 days vs. 10.86 ± 4.69 days, P < 0.001). Total hospitalization costs of VATS lobectomy group and open lobectomy were similar: Renminbi (RMB) 84398.03 ± 13616.13, RMB 81,964.92 ± 16748.11, respectively (P = 0.12). Total non-surgery costs were significantly lower in the VATS lobectomy group than in the open lobectomy group: RMB 41948.40 ± 7747.54 vs. RMB 45752.36 ± 10346.42 (P < 0.001). VATS approach, lung infection, longer post-operative length of stay, health insurance coverage, and lung cancer diagnosis were associated with higher total hospitalization costs (P < 0.05).

**Conclusions:**

VATS lobectomy has a lower lung infection rate, and shorter post-operative LOS than open lobectomy. Future studies are needed to investigate other aspects of clinical effectiveness and the economic burden from a societal perspective.

**Supplementary Information:**

The online version contains supplementary material available at 10.1186/s12962-021-00307-2.

## Background

Lung cancer is the leading cause of cancer-related death worldwide, and China has a relatively high mortality rate compared to most other countries. The incidence rate of lung cancer in 2018 was 18.1% in China, and the death rate due to lung cancer was 24.1% [1]. Lobectomy is a surgical procedure that removes an entire lobe of the lung. This procedure can be performed either through one or few small incisions (minimally invasive) or one long incision (thoracotomy/open lobectomy) [2]. Video-assisted thoracoscopic surgery (VATS) is a type of the minimally invasive thoracic surgery (MITS). It can complete the same task as the traditional thoracotomy and does not require spreading apart the ribs. Compared with traditional open lobectomy, VATS has smaller scars, fewer complications, shorter hospital stay, and less blood loss [3].

Various studies have compared complication rates of open lobectomy and VATS lobectomy. Patients with VATS lobectomy had a significantly lower incidence of short-term complications, a reduced readmission rate, and a shorter length of stay [4–10]. A few studies comparing the economic burden between the two approaches suggested that the VATS lobectomy approach was associated with lower [5, 6, 11] or comparable [7, 8] costs compared to the open lobectomy approach.

In China, the application of MITS, especially VATS lobectomy, among primary lung cancer patients significantly increased from 2.4% in 2008 to 34.4% in 2014 [12]. In 2015, 86.6% of Chinese tertiary hospitals carried out VATS lobectomy, and 73.74% of lung cancer operations in these hospitals adopted the VATS technique [13]. With the rapid adoption of VATS technique in China, consequent outcome assessments are needed to ensure that VATS lobectomy provides equivalent or better outcomes compared with traditional open lobectomy approach. Few studies have examined the Chinese population. In addition, there is a lack of evidence on the economic comparison between the open lobectomy and VATS lobectomy approaches. Thus, this study was to quantify the total medical costs during hospitalization as well as the costs breakdown associated with a lobectomy operation.

The objective of this study was to compare the clinical effectiveness and medical costs of these two existing lobectomy approaches in the Chinese population using real-world data, and to address risk factors associated with total hospitalization costs.

## Methods

### Study population

This retrospective cohort study identified adult patients (> 18 years old) with diagnoses of lung diseases, who were admitted to the Department of Thoracic Surgery, Cancer Hospital of China Medical University, Liaoning Cancer Hospital & Institute for lobectomy in 2018. Inclusion criteria were, (a) patients with general anesthesia for their surgeries; (b) patients who were routinely admitted from outpatient setting; and (c) patients were routinely discharged from the study hospital. Exclusion criteria were (a) patients underwent operations in other organs or systems during the same inpatient admission, (b) patients with incomplete data, or (c) patients with secondary operation during the same hospitalization period. Lung diseases were identified using “diagnosis name” in the EHR. Patients with “diagnosis name” with “lung” were considered as having lung diseases. Furthermore, lung cancer was identified using International Classification of Diseases, Tenth Revision, Clinical Modification (ICD-10-CM) code C34.

### Comparison groups

Two lobectomy approaches were compared, (1) VATS lobectomy, and (2) open lobectomy. Lobectomy approaches were identified based on procedure names in the electronic medical record (EMR). Open lobectomy was defined as with the keywords of “open” AND “lobectomy”. VATS lobectomy was defined as having the keywords of “video-assisted thoracoscopic surgery” AND “lobectomy”.

### Measurement of cost-related clinical outcomes and costs

The EMR was used to collect baseline characteristics and to identify post-operative clinical outcomes and costs. Baseline characteristics included age, gender, health insurance coverage, lung cancer diagnosis status, comorbidities such as hypertension, diabetes, heart disease, and other diseases.

The primary outcomes in this study were cost-related clinical outcomes and hospitalization costs. Cost-related clinical outcomes were measured in terms of blood transfusion rate, lung infection rate, and post-operative length of stay (LOS). Blood transfusion was identified based on the procedures in the EMR and blood transfusion costs at discharge, with the keywords of “transfusion”, or “blood transfusion”. Lung infection was identified as with keywords of “infection” or “pneumonia” in the EMR. Post-operative LOS was measured as the time period from the date of surgery to the date of hospitalization discharge.

Medical costs during hospitalization included general medical service costs, diagnosis costs, non-surgical treatment costs, anesthetic costs, procedure costs, drug costs, blood costs, supply costs for surgery (e.g., stapler costs, cartridge costs, hemostatic material costs, and other supply costs for surgery), supply costs for diagnosis, supply costs for treatment, and other costs (e.g., costs for nursing service or caregivers, and meals) during the hospital stay (cost categories and definitions see Additional file 1: Table S1). Surgery costs were defined as direct medical costs related to operation, including procedure costs, anesthetic costs and supply costs for surgery. We also evaluated the non-surgery costs, which were defined as the total costs excluding anesthetic costs, procedure costs, and supply costs for surgery. For example, a typical item for non-surgery cost category included general medical cost, diagnosis cost, drug cost, and blood cost. We evaluated the direct medical costs from the healthcare system’s perspective.

### Statistical analysis

Propensity score matching (PSM) method was applied, using 1:1 match with a caliper of 0.02 [14]. The propensity scores were calculated by logistic regression. Controlled baseline characteristics included age, sex, health insurance, lung cancer diagnosis, hypertension, diabetes, heart disease and other thoracic disease conditions. The logistic regression estimates for propensity score matching is shown in Additional file 2: Table S2. Descriptive analysis was used to report the baseline characteristics of the study population. Continuous variables were presented as mean ± standard deviation (SD). Categorical variables were reported as counts and percentages. Between-group comparisons were performed. T-tests were used to compare continuous variables, and chi-square tests were used to compare categorical variables. Two-sample Wilcoxon rank-sum (Mann–Whitney) test was used to compare non-normally distributed variables (i.e., post-operative LOS, and costs). Multivariable generalized linear model (GLM) with gamma probability distribution and log-link was used to analyze the factors associated with total costs. Adjusted cost ratio and 95% confidence interval (95% CI) were reported. GLM with gamma distribution can account for significant skewed cost data without the need for retransformation and is the recommended modeling method for cost data in health services research [15, 16]. Significance level was set at two tailed P < 0.05 for all tests. Patients with lung cancer diagnosis were analyzed as a subgroup for above analysis concerned the clinical outcomes and medical costs. Stata 14.0 (StataCorp LLC, College Station, Texas, USA) was used to perform the statistical analysis.

## Results

### Study population

**Fig. 1:**
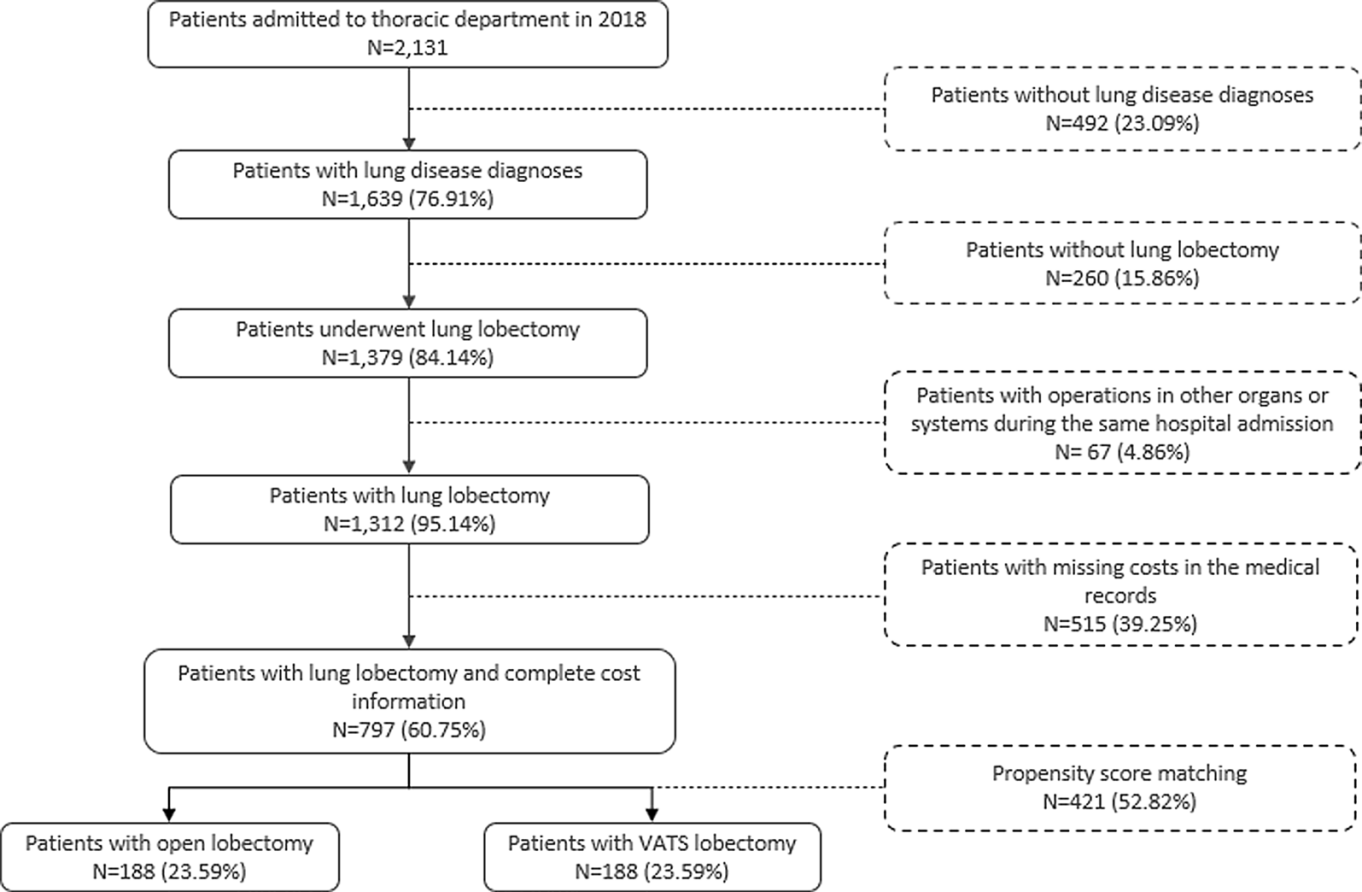
Sample selection flow chart

A total of 2131 patients were admitted to thoracic department in 2018, and 1639 (76.91%) of them were diagnosed with lung disease. After excluding patients without lobectomy, patients who received surgeries in other organs or systems and patients with incomplete data, 797 patients were included in our study. Out of 797 patients studied, 208 (26.10%) patients went through open lobectomy and 589 (73.90%) patients had VATS lobectomy (Fig. 1).

### Patient characteristics

Table 1 shows the patients’ demographic characteristics before and after the PSM. Prior to the PSM, 797 patients were selected based on the inclusion criteria. Only sex was statistically significantly different between the VATS lobectomy and open lobectomy group (p < 0.001). 131 (62.98%) patients in the open lobectomy group were male, compared to 244 (41.43%) patients in the VATS lobectomy group. All the comorbidities were comparable between groups (P > 0.05). Before the PSM, the most common comorbidity in both groups was hypertension (15.38% in open lobectomy group and 19.35% in VATS lobectomy group). Baseline patient characteristics were similar between the selected study population and excluded patients (Additional file 3: Table S3).

**Table 1:** Demographic characteristics for study population

	Before PSM				After PSM							
	Patients with lung disease, n = 797				Patients with lung disease, n = 376				Patients with lung cancer, n = 326			
Baseline characteristics	Overall, n = 797	Open lobectomy, n = 208 (26.10%)	VATS lobectomy, n = 589 (73.90%)	P-value	Overall, n = 376	Open lobectomy, n = 188 (50.00%)	VATS lobectomy, n = 188 (50.00%)	P-value	Overall n = 326	Open lobectomy n = 163 (50.00%)	VATS lobectomy n = 163 (50.00%)	P-value
Age (years), mean ± SD	59.92 ± 8.78	60.23 ± 8.01	59.82 ± 9.04	0.56	59.87 ± 8.16	60.01 ± 7.85	59.72 ± 8.48	0.74	60.34 ± 7.60	60.60 ± 7.39	60.08 ± 7.83	0.54
Age group, n (%)				0.36				0.61				0.67
< 50 years old	100 (12.55)	21 (10.10)	79 (13.41)		34 (9.04)	19 (10.11)	15 (7.98)		24 (7.36)	11 (6.75)	13 (7.98)	
50–59 years old	272 (34.13)	77 (37.02)	195 (33.11)		152 (40.43)	72 (38.30)	80 (42.55)		134 (41.10)	64 (39.26)	70 (42.94)	
≥ 60 years old	425 (53.32)	111 (52.88)	315 (53.48)		190 (50.53)	97 (51.60)	93 (49.47)		168 (51.53)	88 (53.99)	80 (49.08)	
Sex, n (%)				< 0.001				0.83				1.00
Male	375 (47.05)	131 (62.98)	244 (41.43)		223 (59.31)	113 (60.11)	110 (58.51)		195 (59.82)	97 (59.51)	98 (60.12)	
Female	422 (52.95)	77 (37.02)	345 (58.57)		153 (40.69)	75 (39.89)	78 (41.49)		131 (40.18)	66 (40.49)	65 (39.88)	
Health insurance, n (%)				0.68				1.00				1.00
Insured	726 (91.09)	188 (90.38)	538 (91.34)		345 (91.76)	172 (91.49)	173 (92.02)		301 (92.33)	150 (92.02)	151 (92.64)	
Not insured	71 (8.91)	20 (9.62)	51 (8.66)		31 (8.24)	16 (8.51)	15 (7.98)		25 (7.67)	13 (7.98)	12 (7.36)	
Primary diagnosis, n (%)				0.17				1.00				NA
Lung cancer	696 (87.33)	176 (84.62)	520 (88.29)		334 (88.83)	167 (88.83)	167 (88.83)		326 (100.00)	163 (100.00)	163 (100.00)	
Other lung diseases	101 (12.67)	32 (15.38)	69 (11.71)		42 (11.17)	21 (11.17)	21 (11.17)		0	0	0	
Comorbidity, n (%)												
Hypertension				0.20				0.89				1.00
Yes	146 (18.32)	32 (15.38)	114 (19.35)		56 (14.89)	29 (15.42)	27 (14.36)		41 (12.58)	20 (12.27)	21 (12.88)	
No	651 (81.68)	176 (84.62)	475 (80.65)		320 (85.11)	159 (84.58)	161 (85.64)		285 (87.42)	143 (87.73)	142 (87.12)	
Diabetes				0.66				1.00				0.71
Yes	75 (9.41)	18 (8.65)	57 (9.68)		36 (9.57)	18 (9.57)	18 (9.57)		31 (9.51)	14 (8.59)	17 (10.43)	
No	722 (90.59)	190 (91.35)	532 (90.32)		340 (90.43)	170 (90.43)	170 (90.43)		295 (90.49)	149 (91.41)	146 (89.57)	
Heart disease				0.27				0.25				1.00
Yes	64 (8.03)	13 (6.25)	51 (8.66)		20 (5.32)	13 (6.91)	7 (3.72)		16 (4.91)	8 (4.91)	8 (4.91)	
No	733 (91.97)	195 (93.75)	538 (91.34)		356 (94.68)	175 (93.09)	181 (96.28)		310 (95.09)	155 (95.09)	155 (95.09)	
Other diseases				0.77				0.83				0.62
Yes	54 (6.78)	15 (7.21)	39 (6.62)		24 (6.38)	11 (5.85)	13 (6.91)		17 (5.21)	10 (6.14)	7 (4.29)	
No	743 (93.22)	193 (92.79)	550 (93.38)		352 (93.62)	177 (94.15)	175 (93.09)		309 (94.79)	153 (93.86)	156 (95.71)	

*PSM* propensity score matching, *SD* standard deviation

After the PSM, 376 patients were included in the analytic sample. The average age in the overall sample was 59.87 ± 8.16 years old. 345 (91.76%) patients had health insurance coverage. 334 (88.83%) patients had lung cancer diagnosis. Overall, 56 (14.89%) patients had hypertension, as the most common comorbidity among the sample (Table 1).

### Cost-related clinical outcomes

Three cost-related clinical outcomes assessed in this study were (1) blood transfusion rate, (2) lung infection rate, and (3) post-operative LOS (Table 2). Overall, 10 patients had blood transfusion post operation. The open lobectomy group (n = 6, 3.20%) had a similar blood transfusion rate, compared with the VATS lobectomy group (n = 4, 2.10%) (P = 0.75). One hundred fifteen (115) patients experienced post-operative lung infections in the study sample. Patients with open lobectomy (n = 75, 39.89%) had a higher lung infection rate post operation than patients in the VATS lobectomy group (n = 40, 21.28%) (P < 0.001). On average, the post-operative LOS for all patients was 10.14 ± 4.08 days. A longer post-operative LOS was observed in patients with open lobectomy, with a mean of 10.86 ± 4.69 days. For patients who underwent the VATS lobectomy, the average post-operative LOS was 9.42 ± 3.22 days. The difference was statistically significant between these two groups (P < 0.001).

**Table 2:** Cost related clinical outcomes and hospitalization costs comparisons between groups

Clinical outcomes	Patients with lung disease				Patients with lung cancer			
	Overall, n = 376	Open lobectomy, n = 188	VATS lobectomy, n = 188	P-value	Overall, n = 326	Open lobectomy, n = 163	VATS lobectomy, n = 163	P-value
Blood transfusion, n (%)	10 (2.66)	6 (3.19)	4 (2.13)	0.75	10 (3.07)	4 (2.45)	6 (3.68)	0.75
Lung infection, n (%)	115 (30.59)	75 (39.89)	40 (21.28)	< 0.001	95 (29.14)	64 (39.26)	31 (19.02)	< 0.001
Post-operative LOS (days), mean ± SD	10.14 ± 4.08	10.86 ± 4.69	9.42 ± 3.22	< 0.01	10.19 ± 4.25	11.06 ± 4.91	9.32 ± 3.25	< 0.001
Total hospitalization costs (RMB), mean ± SD	83181.47 ± 15090.94	81964.92 ± 16748.11	84398.03 ± 13161.13	0.12	84139.26 ± 15170.14	82848.46 ± 16928.87	85430.05 ± 13105.24	0.13
Total non-surgery costs (RMB), mean ± SD	43850.38 ± 9324.20	45752.36 ± 10346.42	41948.40 ± 7747.54	< 0.001	44520.89 ± 9474.80	46633.03 ± 10538.43	42408.75 ± 7749.95	< 0.001

*LOS* length of stay, *SD* standard deviation

### Total hospitalization costs and cost breakdown

VATS lobectomy and open lobectomy did not differ in the total hospitalization costs (RMB 84398.03 ± 13161.13 vs. RMB 81964.92 ± 16748.11, P = 0.12) (Table 2). Non-surgery costs were significantly lower for VATS lobectomy than open lobectomy (RMB 41948.40 ± 7747.54 vs. RMB 45752.36 ± 10346.42, P < 0.001).

**Fig. 2:**
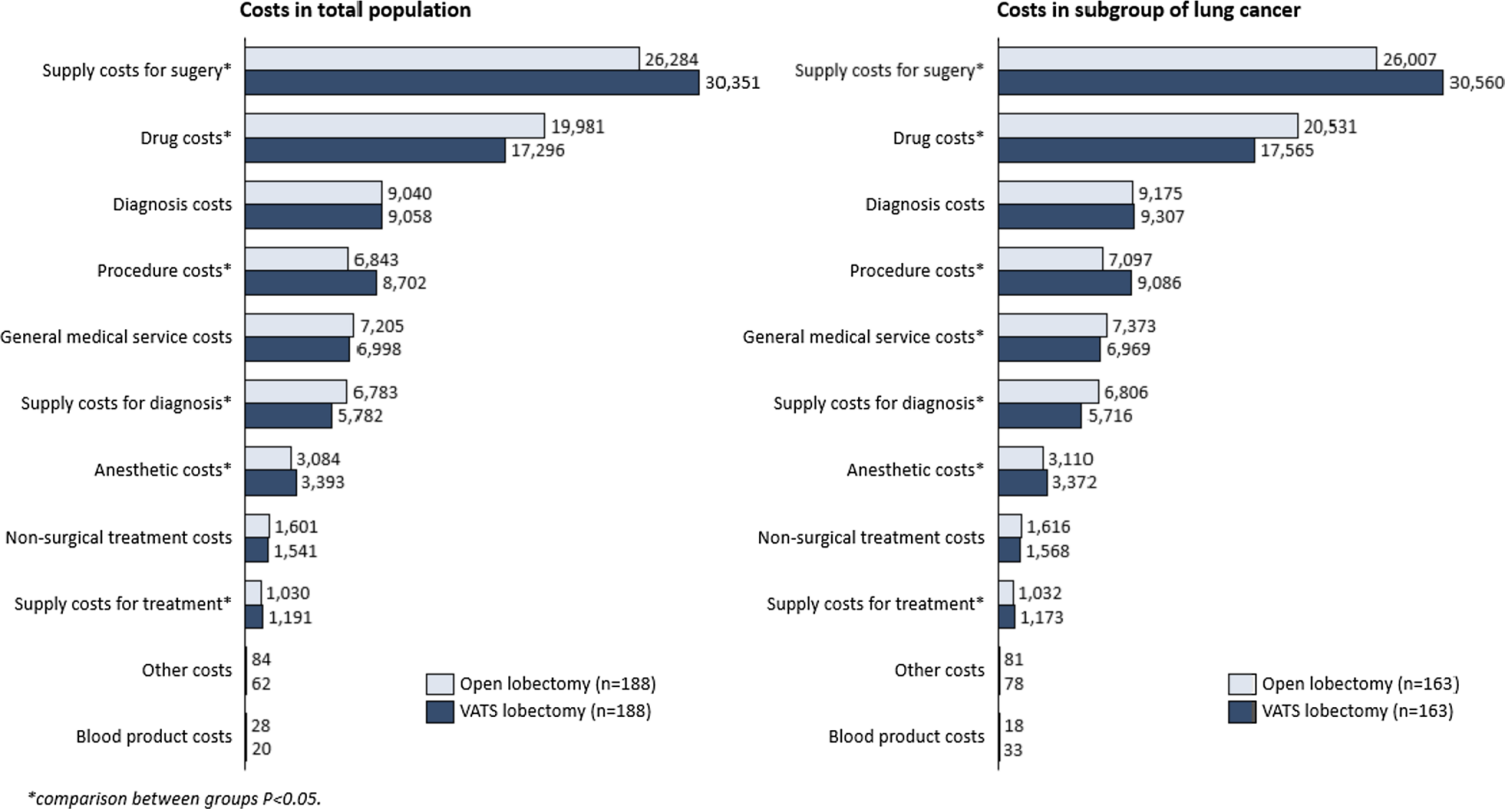
Cost breakdown comparisons between VATS and open lobectomy procedures

Figure 2 and Additional file 4: Table S4 present the hospitalization cost breakdown by cost categories. In all categories of cost breakdown, supply costs for surgery were the biggest driver of the total hospitalization costs, and it was significantly higher for VATS lobectomy than open lobectomy (RMB 30350.94 ± 8229.41 in VATS lobectomy vs. RMB 26283.92 ± 11070.10 in open lobectomy, P < 0.001). Among the supply costs for surgery, cartridge costs were significantly higher in VATS lobectomy group, while hemostatic material costs in VATS group was significantly lower (Additional file 5: Table S5). The second biggest cost driver for both groups was drug costs, and it was significantly lower for VATS lobectomy than open lobectomy (RMB 17296.26 ± 5294.47 in VATS lobectomy vs. RMB 19981.01 ± 6984.33 in open lobectomy, P < 0.001).

### Associated factors of total hospitalization costs

Lobectomy approach types, baseline characteristics and clinical outcomes were included in the GLM regression to further evaluate their impacts on the total hospitalization costs (Table 3). Age group was used in the model instead of using age as a continuous variable.

**Table 3:** Multivariable generalized linear model for total hospitalization costs

	Patients with lung disease, n = 376			Patients with lung cancer, n = 326		
	Adjusted cost ratio	95% CI	P value	Adjusted cost ratio	95% CI	P value
Lobectomy approach (reference: open lobectomy)	1.09	(1.06, 1.13)	< 0.001	1.10	(1.06, 1.14)	< 0.001
Blood transfusion (reference: no)	1.07	(0.97, 1.18)	0.15	1.03	(0.93, 1.13)	0.62
Lung infection (reference: no)	1.18	(1.14, 1.22)	< 0.001	1.19	(1.15, 1.24)	< 0.001
Post-operative length of stay	1.01	(1.01, 1.02)	< 0.001	1.01	(1.01, 1.02)	< 0.001
Age group (reference: < 50 years old)						
50–59 years old	1.06	(1.00, 1.13)	0.04	1.06	(0.99, 1.13)	0.10
≥ 60 years old	1.05	(0.99, 1.12)	0.08	1.07	(1.00, 1.14)	0.07
Sex (reference: female)	1.03	(1.00, 1.06)	0.07	1.03	(0.99, 1.06)	0.12
Health insurance (reference: not insured)	1.08	(1.02, 1.14)	0.01	1.01	(0.95, 1.08)	0.75
Lung cancer diagnosis (reference: no)	1.11	(1.05, 1.16)	< 0.001	NA	NA	NA
Hypertension (reference: no)	1.02	(0.97, 1.06)	0.51	1.02	(0.96, 1.07)	0.55
Diabetes (reference: no)	1.04	(0.98, 1.09)	0.19	1.04	(0.98, 1.10)	0.19
Heart disease (reference: no)	1.05	(0.98, 1.12)	0.19	1.1	(1.02, 1.19)	0.02
Other thoracic diseases (reference: no)	0.98	(0.92, 1.05)	0.64	0.99	(0.91, 1.07)	0.74

*95% CI* 95% confidence interval

In the overall study population, after controlling for covariates, the VATS lobectomy approach was significantly associated with higher total hospitalization costs. Compared to open lobectomy, the VATS lobectomy approach increased the total hospitalization costs by 9% (coefficient = 1.09, 95% CI 1.06, 1.13. P < 0.001). Lung infection post operation also increased the total hospitalization costs by 1.18 times (95% CI 1.14, 1.22. P < 0.001), compared with patients without lung infection. An additional day of post-operative LOS increased the total hospitalization costs by 1.01 times (95% CI 1.01, 1.02. P < 0.001).

Patients aged between 50 to 59 years old had the highest total hospitalization costs, and its impact on the hospitalization costs was significant (P = 0.04). Controlling for other covariates, health insurance coverage increased the total hospitalization costs by 1.08 times (95% CI 1.02, 1.14. P = 0.01). Additionally, the total hospitalization costs for patients with lung cancer diagnosis was 1.11 times (95% CI 1.05, 1.16. P < 0.001) higher than patients without lung cancer diagnosis.

### Subgroup analyses

*Demographic characteristics* In the matched sample, of the 326 patients with lung cancer, 163 (50%) patients had open lobectomy and the other 163 (50%) patients had VATS lobectomy (Table 1). The characteristics of the subgroup were comparable with the overall study sample.

*Post-operative outcomes* Among 326 patients with lung cancer, 10 patients had blood transfusion with 4 (2.45%) patients from the open lobectomy group, and 6 (3.68%) patients from the VATS lobectomy group. The difference in blood transfusion between open lobectomy and VATS lobectomy group was not significant in this subgroup (P = 0.75). The VATS group had a lower lung infection rate (19.02% vs. 39.26%, P < 0.001) and a shorter LOS (9.32 ± 3.25 vs. 11.06 ± 4.91, P < 0.001), compared to the open lobectomy group (Table 2).

*Hospitalization costs and cost breakdown* In this subgroup, the mean difference in total hospitalization costs between open lobectomy and VATS lobectomy was not significant (RMB 82848.46 ± 16928.87 in the open lobectomy group vs. RMB 85430.05 ± 13105.24 in the VATS lobectomy group, P = 0.13) (Table 2). Non-surgery costs were significantly lower for VATS lobectomy than open lobectomy (RMB 42408.75 ± 7749.95 in VATS lobectomy vs. RMB 46633.03 ± 10538.43 in open lobectomy, P < 0.001). Diagnosis costs, treatment costs, blood product costs and other costs remained equivalent between the two lobectomy groups (P > 0.05). Other types of costs remained significantly different between the open lobectomy group and VATS lobectomy group (P < 0.05) (Fig. 2).

*Associated factors of total hospitalization costs* Undergoing VATS lobectomy, having lung infection, longer post-operative LOS, and having heart disease were positively associated with total hospitalization costs P < 0.05) (Table 3).

## Discussion

To our best knowledge, this was the first study comparing the post-operative outcomes and costs with the most comprehensive cost analysis between VATS lobectomy and open lobectomy among Chinese patients with lung diseases, regardless of lung cancer status. The post-operative outcomes of VATS lobectomy, including lung infection rate and post-operative LOS, were significantly better than open lobectomy. This was also the first study assessing risk factors for high hospitalization costs of lobectomy operation in the Chinese population. Overall, total hospitalization costs among the patients with VATS lobectomy were comparable with open lobectomy. However, the total non-surgery costs were significantly lower in VATS compared to open lobectomy. This may be due to the lower complication rates found in the study population, as well as better intraoperative and postoperative clinical outcomes, the drug costs, costs for blood transfusion and non-surgical treatment costs were significantly lower in the VATS group.

Our findings were consistent with the previous studies [4, 7, 9, 17, 18]. First, post-operative clinical outcomes including lung infection, and post-operative LOS were all better in the VATS lobectomy group, compared to the open lobectomy group. Second, the procedure costs, cartridge costs for VATS lobectomy were significantly higher, and may be due to the advanced technology of VATS lobectomy. Blood costs, drug costs and hemostatic material costs in the open lobectomy group were higher, and it might result from a relatively greater trauma from open lobectomy approach. A prior study also found hospitalization costs in the VATS lobectomy group were significantly higher due to higher operative and instrument costs, compared with the open lobectomy approach [18]. The GLM regression results showed that the total hospitalization costs were associated with post-operative lung infection, post-operative LOS, sex, and lung cancer diagnosis status.

Besides lung infection and blood transfusion, hospital readmission due to surgical complications in the study population was investigated. However, no hospitalization due to the complications or failure of operation was found in either VATS or open lobectomy group. Thus, it was not reported in the clinical outcomes.

Long-term survival from these two approaches was also evaluated in the previous studies. Most study findings showed the long-term survival was comparable between open lobectomy and VATS lobectomy [19–24]. We did not include this as a clinical outcome in our study. The main reason was that even either VATS or open lobectomy could lead to blood loss, infection, and physical pain, significant bleeding during lung resection surgery was found to be rare in a retrospective matched cohort analysis using real-world data [25]. Thus, we assume the complications such as bleeding or lung infection from either lobectomy approach would not significantly increase the chance of death from the procedure.

In our study population, before the PSM, patients in open lobectomy group and VATS lobectomy group were similar, except for the sex distribution. More males received open lobectomy, while more females received VATS lobectomy. It may be because more males were smokers, with worse pulmonary function, and with advanced lung cancer. Thus, open lobectomy might be more appropriate in this situation, as it would be safer and more likely to remove the whole tumor [26].

There were some limitations in our study. First, either open lobectomy or VATS lobectomy requires surgeons to have sufficient training and experience, and it plays an important role in the assessment of complications and hospitalization costs, as the economic impact could be magnified as the surgeons’ experience increases [5]. Thus, without the consideration of surgeons’ experience, the interpretation of the comparison between open lobectomy and VATS lobectomy might be biased. Second, this study used medical records for a single hospital. Due to the unbalanced development of the thoracic surgery technology in different regions in China, the study population may not be representative for the other regions of China. Third, due to a lack of access to smoking history, pulmonary function, cancer stage, and adjunctive treatment, these baseline characteristics were not controlled for in the PSM. These factors may have confounded the results. Fourth, due to a lack of information of ICU stay, the LOS was not differentiated between ICU admission and ward hospitalization. The length of the lobectomy operation for each group was not available, thus, when reporting the other costs, the opportunity cost of operation between the two techniques was not able to be considered. Fifth, PSM can only match on the observables. It cannot manage the differences in unobserved variables that still introduce selection bias. Lastly, readmission which could be an important post-operative complication was not considered as a clinical outcome in this study because no cases were identified in the EHR. All the patients were usually followed up in outpatient settings. A lack of readmission may have contributed to the relatively lower non-surgery costs with the VATS approach.

The findings of this study can provide the patients, physicians, and health caregivers with a view of the clinical effectiveness and economic burden of each approach. It can also help policy-makers to make informed decisions to improve healthcare outcomes at both the individual and population levels. More assessments are still needed in the future.

## Conclusions

Our study suggests that from the health system’s perspective, the utilization of the VATS lobectomy approach led to higher hospitalization costs. However, these direct procedure costs were offset by reductions in post-operative blood transfusion rate, lung infection rate and hospital LOS, compared with the open lobectomy approach. A more comprehensive and prospective comparison is needed to include patient-reported outcomes, as well as to assess it from the societal perspective.

## Supplementary Information

**Additional file 1:Table S1.** Cost categories and definitions.

**Additional file 2:Table S2.** Logistic regression estimates for propensity score matching.

**Additional file 3:Table S3.** Sensitivity analysis of baseline characteristics comparison between included population and missing population.

**Additional file 4:Table S4.** Hospitalization costs comparisons between groups.

**Additional file 5:Table S5.** Cost analysis of supply costs for surgery.

## Data Availability

The data that support the findings of this study are available from the corresponding author upon reasonable request.

## Abbreviations

VATS: Video-Assisted Thoracoscopic Surgery
LOS: Length of stay
MITS: Minimally invasive thoracic surgery
EMR: Electronic medical record
PSM: Propensity score matching
SD: Standard deviation
GLM: Generalized linear model
CI: Confidence interval
RMB: Renminbi
