# Supplementary material for: Comparison of cost effectiveness between video-assisted thoracoscopic surgery (vats) and open lobectomy: a retrospective study
Source: Cost Eff Resour Alloc. 2021 Aug 28;19:55. doi: 10.1186/s12962-021-00307-2 (PMC8400899; doi:10.1186/s12962-021-00307-2)
Supplement: Supplementary file 1 — Additional file 1:Table S1. Cost categories and definitions. [file 12962_2021_307_MOESM1_ESM.docx]

**Supplementary Table 1. Cost categories and definitions**

| **Cost** | **Definition** | **Surgery cost** | **Non-Surgery Cost** |
| --- | --- | --- | --- |
|  |  |  |  |
| General medical service cost | Service fee for basic diagnosis and examination, cost of room and board, cost of nursing |  | x |
| Diagnosis cost | Cost of imagological diagnosis, pathological diagnosis, lab tests, and other diagnosis procedure during index hospitalization |  | x |
| Non-surgical treatment cost | Cost of treatment operation (exclude surgical treatment) during index hospitalization |  | x |
| Anesthesia cost | Cost of anesthesia during operation | x |  |
| Procedure cost | Cost of surgical operation during index hospitalization | x |  |
| Drug cost | Cost of medication during index hospitalization |  | x |
| Blood cost | Cost of blood products, (e.g., plasma, red blood cells, platelets, whole blood, etc.) |  | x |
| Supply costs for diagnosis | Cost of separately charged supply used for diagnosis during index hospitalization |  | x |
| Supply costs for treatment | Cost of separately charged supply used for treatment during index hospitalization |  | x |
| Supply costs for surgery | Cost of separately charged supply used for surgery during index hospitalization | x |  |
| Other | Other cost during index hospitalization (e.g., cost of meals). |  | x |
