# Supplementary material for: Comparison of cost effectiveness between video-assisted thoracoscopic surgery (vats) and open lobectomy: a retrospective study
Source: Cost Eff Resour Alloc. 2021 Aug 28;19:55. doi: 10.1186/s12962-021-00307-2 (PMC8400899; doi:10.1186/s12962-021-00307-2)
Supplement: Supplementary file 2 — Additional file 2:Table S2. Logistic regression estimates for propensity score matching. [file 12962_2021_307_MOESM2_ESM.docx]

**Supplementary table 1. Logistic regression estimates for propensity score matching**

|  | PSM for overall population | | | | PSM for lung cancer population | | | |
| --- | --- | --- | --- | --- | --- | --- | --- | --- |
|  | Coefficients | Standard Error | Z value | P value | Coefficients | Standard Error | Z value | P value |
| (Intercept) | 1.4 | 0.62 | 2.253 | 0.024 | 1.9 | 0.69 | 2.761 | 0.006 |
| age | -0.01 | 0.01 | -0.673 | 0.501 | -0.01 | 0.01 | -0.865 | 0.387 |
| gender (male) | -0.89 | 0.17 | -5.225 | <0.001 | -0.82 | 0.18 | -4.47 | <0.001 |
| insurance (insured) | 0.14 | 0.29 | 0.505 | 0.613 | 0.1 | 0.31 | 0.323 | 0.746 |
| Lung cancer (diagnosed with lung cancer) | 0.33 | 0.24 | 1.364 | 0.173 |  |  |  |  |
| hypertension (diagnosed with hypertension) | 0.23 | 0.24 | 0.966 | 0.334 | 0.35 | 0.26 | 1.385 | 0.166 |
| diabetes (diagnosed with diabetes) | 0.29 | 0.3 | 0.982 | 0.326 | 0.05 | 0.33 | 0.167 | 0.868 |
| heart disease (diagnosed with heart disease) | 0.27 | 0.34 | 0.788 | 0.431 | 0.41 | 0.36 | 1.128 | 0.259 |
| other disease (diagnosed with other disease) | 0.04 | 0.32 | 0.135 | 0.893 | -0.12 | 0.35 | -0.333 | 0.739 |
| *PSM: propensity score matching* | | | | | | | | |
