# Supplementary material for: Comparison of cost effectiveness between video-assisted thoracoscopic surgery (vats) and open lobectomy: a retrospective study
Source: Cost Eff Resour Alloc. 2021 Aug 28;19:55. doi: 10.1186/s12962-021-00307-2 (PMC8400899; doi:10.1186/s12962-021-00307-2)
Supplement: Supplementary file 3 — Additional file 3:Table S3. Sensitivity analysis of baseline characteristics comparison between included population and missing population. [file 12962_2021_307_MOESM3_ESM.doc]

**Supplementary table 2. Sensitivity analysis of baseline characteristics comparison between included population and missing population.**

| Characteristics | Included population  n=797 | Missing population  n=515 | P-value |
| --- | --- | --- | --- |
| Operative approach, n (%) |  |  | 0.013 |
| VATS | 589 (73.90%) | 348 (67.57%) |  |
| Open | 208 (26.10%) | 167 (32.43%) |  |
| Age (years), mean ± SD | 59.92 ± 8.78 | 60.13 ± 8.64 | 0.410 |
| Gender, n (%) |  |  | 0.550 |
| Male | 375 (47.05%) | 251 (48.74%) |  |
| Female | 422 (52.95%) | 264 (51.26%) |  |
| Health insurance, n (%) |  |  | 0.394 |
| Insured | 726 (91.09%) | 476 (92.43%) |  |
| Not insured | 71 (8.91%) | 39 (7.57%) |  |
| Primary diagnosis, n (%) |  |  | 0.896 |
| Lung cancer | 696 (87.33%) | 451 (87.57%) |  |
| Other lung diseases | 101 (12.67%) | 64 (12.43%) |  |
| ***Comorbidities*** |  |  |  |
| Hypertension, n (%) |  |  | 0.305 |
| Yes | 146 (18.32%) | 83 (16.12%) |  |
| No | 651 (81.68%) | 432 (83.88%) |  |
| Diabetes, n (%) |  |  | 0.950 |
| Yes | 75 (9.41%) | 49 (9.51%) |  |
| No | 722 (90.59%) | 466 (90.49%) |  |
| Heart disease, n (%) |  |  | 0.098 |
| Yes | 64 (8.03%) | 29 (5.63%) |  |
| No | 733 (91.97%) | 486 (94.37%) |  |
| Other diseases, n (%) |  |  | 0.880 |
| Yes | 54 (6.78%) | 36 (6.99%) |  |
| No | 743 (93.22%) | 479 (93.01%) |  |
| *SD: Standard Deviation.* |  |  |  |
