# Supplementary material for: Comparison of cost effectiveness between video-assisted thoracoscopic surgery (vats) and open lobectomy: a retrospective study
Source: Cost Eff Resour Alloc. 2021 Aug 28;19:55. doi: 10.1186/s12962-021-00307-2 (PMC8400899; doi:10.1186/s12962-021-00307-2)
Supplement: Supplementary file 4 — Additional file 4:Table S4. Hospitalization costs comparisons between groups. [file 12962_2021_307_MOESM4_ESM.docx]

**Supplementary Table 3. Hospitalization costs comparisons between groups**

|  | **Patients with lung disease** | | | | **Patients with lung cancer** | | | |
| --- | --- | --- | --- | --- | --- | --- | --- | --- |
|  | **Overall  n=376** | **Open lobectomy n=188** | **VATS lobectomy  n=188** | **P-value** | **Overall  n=326** | **Open lobectomy  n=163** | **VATS lobectomy  n=163** | **P-value** |
| **Total costs** |  |  |  | 0.12 |  |  |  | 0.13 |
| Mean±SD | 83181.47 ± 15090.94 | 81964.92 ± 16748.11 | 84398.03 ± 13161.13 |  | 84139.26 ± 15170.14 | 82848.46 ± 16928.87 | 85430.05 ± 13105.24 |  |
| Median (IQR) | 82933.68 (73310.92-92880.75) | 81412.30 (69020.73-93924.36) | 83581.92 (76242.64-91609.68) |  | 83320.82 (73854.16-93782.05) | 81947.12 (69251.43-94984.76) | 84096.33 (77730.80-91523.70) |  |
| **General medical service costs** |  |  |  | 0.23 |  |  |  | **0.03** |
| Mean±SD | 7101.42 ± 1651.46 | 7204.94 ± 1701.16 | 6997.90 ± 1598.05 |  | 7171.01 ± 1691.99 | 7373.35 ± 1751.65 | 6968.68 ± 1610.18 |  |
| Median (IQR) | 6765.88 (5958.84-7834.19) | 6848.54 (6196.19-7916.94) | 6757.89 (5824.84-7767.34) |  | 6794.99 (6013.94-7906.14) | 6966.74 (6284.70-8103.84) | 6743.23 (5857.24-7678.44) |  |
| **Diagnosis costs** |  |  |  | 0.95 |  |  |  | 0.68 |
| Mean±SD | 9048.99 ± 2837.80 | 9039.93 ± 3126.30 | 9058.05 ± 2524.95 |  | 9240.91 ± 2908.59 | 9175.26 ± 3129.02 | 9306.55 ± 2678.16 |  |
| Median (IQR) | 8556.35 (7318.63-10178.30) | 8544.96 (7024.49-10182.31) | 8647.59 (7499.69-10178.30) |  | 8613.33 (7518.83-10289.95) | 8595.40 (7282.77-10489.49) | 8740.27 (7658.86-10252.88) |  |
| **Treatment costs** |  |  |  | 0.11 |  |  |  | 0.21 |
| Mean±SD | 1571.06 ± 362.15 | 1601.23 ± 392.84 | 1540.90 ± 326.88 |  | 1592.00 ± 353.36 | 1616.47 ± 368.81 | 1567.53 ± 336.57 |  |
| Median (IQR) | 1458.60 (1361.80-1859.55) | 1458.60 (1386.00-2007.77) | 1386.00 (1337.60-1788.60) |  | 1458.60 (1386.00-2022.35) | 1458.60 (1386.00-1993.20) | 1386.00 (1349.70-2041.60) |  |
| **Anesthetic costs** |  |  |  | **<0.001** |  |  |  | **<0.001** |
| Mean±SD | 3238.62 ± 461.63 | 3083.77 ± 439.53 | 3393.47 ± 431.22 |  | 3240.94 ± 481.85 | 3109.70 ± 455.43 | 3372.17 ± 473.00 |  |
| Median (IQR) | 3186.15 (2912.80-3498.55) | 3026.10 (2769.80-3269.47) | 3360.50 (3106.95-3626.15) |  | 3177.35 (2897.40-3502.40) | 3040.40 (2799.50-3316.50) | 3304.40 (3028.85-3633.30) |  |
| **Procedure costs** |  |  |  | **<0.001** |  |  |  | **<0.001** |
| Mean±SD | 7772.31 ± 2247.73 | 6842.62 ± 1606.02 | 8701.99 ± 2411.30 |  | 8091.26 ± 2250.66 | 7096.89 ± 1441.99 | 9085.62 ± 2468.43 |  |
| Median (IQR) | 7502.00 (6677.00-8767.00) | 6732.00 (5907.00-7447.00) | 8767.00 (8712.00-9097.00) |  | 8643.80 (6732.00-9097.00) | 6908.00 (5962.00-7502.00) | 8767.00 (8767.00-9097.00) |  |
| **Drug costs** |  |  |  | **<0.001** |  |  |  | **<0.001** |
| Mean±SD | 18638.64 ± 6333.28 | 19981.01 ± 6984.33 | 17296.26 ± 5294.47 |  | 19047.81 ± 6431.93 | 20530.77 ± 7226.10 | 17564.86 ± 5133.49 |  |
| Median (IQR) | 18285.23 (14398.27-21566.99) | 19461.97 (15595.10-22757.50) | 16803.12 (13653.20-20190.75) |  | 18583.04 (14909.08-21830.10) | 19911.47 (16118.49-23962.47) | 16933.36 (14318.63-20209.62) |  |
| **Blood costs** |  |  |  | 0.65 |  |  |  | 0.43 |
| Mean±SD | 24.17 ± 182.66 | 28.41 ± 187.97 | 19.93 ± 177.59 |  | 25.74 ± 176.23 | 17.98 ± 131.11 | 33.49 ± 212.12 |  |
| Median (IQR) | 0.00 (0.00-0.00) | 0.00 (0.00-0.00) | 0.00 (0.00-0.00) |  | 0.00 (0.00-0.00) | 0.00 (0.00-0.00) | 0.00 (0.00-0.00) |  |
| **Supply costs for diagnosis** |  |  |  | **<0.001** |  |  |  | **<0.001** |
| Mean±SD | 6282.35 ± 1457.54 | 6782.71 ± 1424.40 | 5781.98 ± 1314.48 |  | 6261.13 ± 1459.96 | 6805.99 ± 1449.65 | 5716.27 ± 1255.88 |  |
| Median (IQR) | 6164.33 (5074.55-7277.97) | 6738.02 (5765.76-7634.99) | 5556.24 (4775.51-6687.69) |  | 6148.21 (5073.24-7286.05) | 6822.74 (5700.41-7672.28) | 5539.07 (4777.69-6536.38) |  |
| **Supply costs for treatment** |  |  |  | **<0.001** |  |  |  | **<0.001** |
| Mean±SD | 1110.62 ± 258.57 | 1030.21 ± 213.46 | 1191.03 ± 274.78 |  | 1102.48 ± 250.88 | 1031.93 ± 202.07 | 1173.03 ± 274.64 |  |
| Median (IQR) | 1030.55 (934.55-1285.60) | 946.55 (934.55-1054.55) | 1100.75 (934.55-1352.45) |  | 1006.55 (934.55-1204.35) | 958.55 (934.55-1054.55) | 1078.55 (934.55-1352.45) |  |
| **Supply costs for surgery** |  |  |  | **<0.001** |  |  |  | **<0.001** |
| Mean±SD | 28317.43 ± 9951.27 | 26283.92 ± 11070.10 | 30350.94 ± 8229.41 |  | 28283.61 ± 10158.34 | 26007.01 ± 10924.76 | 30560.21 ± 8788.67 |  |
| Median (IQR) | 28173.83 (21985.08-35051.75) | 24176.63 (16882.83-37142.60) | 29332.25 (25379.83-34279.57) |  | 27799.80 (21950.75-34778.00) | 23957.00 (16810.60-36344.65) | 29276.00 (25041.50-34778.00) |  |
| **Other costs** |  |  |  | 0.08 |  |  |  | 0.83 |
| Mean±SD | 73.13 ± 117.72 | 83.91 ± 124.04 | 62.34 ± 110.31 |  | 79.82 ± 122.22 | 81.29 ± 111.03 | 78.34 ± 132.79 |  |
| Median (IQR) | 36.00 (0.00-120.00) | 54.00 (0.00-129.00) | 15.00 (0.00-102.00) |  | 60.00 (0.00-126.00) | 60.00 (0.00-132.00) | 60.00 (0.00-120.00) |  |
| *SD: Standard Deviation; IQR: Interquartile Range.* | | | | | | | | |
