# Supplementary material for: Comparison of cost effectiveness between video-assisted thoracoscopic surgery (vats) and open lobectomy: a retrospective study
Source: Cost Eff Resour Alloc. 2021 Aug 28;19:55. doi: 10.1186/s12962-021-00307-2 (PMC8400899; doi:10.1186/s12962-021-00307-2)
Supplement: Supplementary file 5 — Additional file 5:Table S5. Cost analysis of supply costs for surgery. [file 12962_2021_307_MOESM5_ESM.docx]

**Supplementary Table 4. Cost analysis of supply costs for surgery**

| **cost breakdown of supply costs for surgery** | **Patients with lung disease** | | | |  | **Patients with lung cancer** | | |
| --- | --- | --- | --- | --- | --- | --- | --- | --- |
|  | **Overall  n=376** | **Open lobectomy n=188** | **VATS lobectomy n=188** | **P-value** | **Overall  n=326** | **Open lobectomy n=163** | **VATS lobectomy n=163** | **P-value** |
| **Stapler costs** |  |  |  | **<0.001** |  |  |  | **<0.001** |
| Mean±SD | 7152.67 ± 2864.57 | 8067.94 ± 3597.20 | 6237.40 ± 1353.22 |  | 7110.90 ± 2887.78 | 8054.30 ± 3588.50 | 6167.49 ± 1435.88 |  |
| Median (IQR) | 6930.00 (5124.00-7140.00) | 7140.00 (5124.00-11502.95) | 6930.00 (5124.00-6930.00) |  | 6930.00 (5124.00-7140.00) | 7140.00 (5124.00-11151.20) | 6930.00 (5124.00-7140.00) |  |
| **Cartridge costs** |  |  |  | **<0.001** |  |  |  | **<0.001** |
| Mean±SD | 12610.25 ± 7930.45 | 10053.00 ± 7642.97 | 15167.49 ± 7386.24 |  | 12811.83 ± 8163.59 | 9919.47 ± 7451.28 | 15704.18 ± 7832.21 |  |
| Median (IQR) | 13125.00 (7875.00-18270.00) | 10500.00 (2381.40-15750.00) | 13125.00 (10500.00-18375.00) |  | 13125.00 (7875.00-18270.00) | 10500.00 (2381.40-15750.00) | 15750.00 (10500.00-20212.50) |  |
| **Hemostatic material costs** |  |  |  | **<0.001** |  |  |  | **0.001** |
| Mean±SD | 4966.78 ± 2574.19 | 5496.74 ± 3214.84 | 4436.82 ± 1545.47 |  | 4866.77 ± 2513.75 | 5309.78 ± 3179.46 | 4423.76 ± 1474.11 |  |
| Median (IQR) | 4574.90 (3307.50-5863.80) | 4951.85 (3179.95-8742.40) | 4237.85 (3310.20-5752.00) |  | 4574.90 (3310.70-5752.00) | 4710.40 (3148.00-8017.90) | 4469.90 (4041.50-5752.00) |  |
| **Other supply costs for surgery** |  |  |  | **<0.001** |  |  |  | **<0.001** |
| Mean±SD | 3518.89 ± 1493.64 | 2615.53 ± 1057.18 | 4422.26 ± 1309.71 |  | 3441.47 ± 1374.73 | 2664.95 ± 1070.59 | 4217.98 ± 1196.62 |  |
| Median (IQR) | 3353.75 (2377.65-4462.35) | 2390.80 (2057.40-3016.27) | 4332.90 (3579.90-5203.10) |  | 3313.58 (2394.10-4326.20) | 2430.15 (2088.30-3096.30) | 4120.65 (3460.60-5086.00) |  |
| *SD: Standard Deviation; IQR: Interquartile Range.* | | | | | | | | |
